# Post-transcriptional regulation during stress

**DOI:** 10.1093/femsyr/foac025

**Published:** 2022-05-13

**Authors:** Mariana Hernández-Elvira, Per Sunnerhagen

**Affiliations:** Department of Chemistry and Molecular Biology, Lundberg Laboratory, University of Gothenburg, PO Box 462, S-405 30 Göteborg, Sweden; Department of Chemistry and Molecular Biology, Lundberg Laboratory, University of Gothenburg, PO Box 462, S-405 30 Göteborg, Sweden

**Keywords:** RNA, biology, *Saccharomyces cerevisiae*, *Schizosaccharomyces pombe*

## Abstract

To remain competitive, cells exposed to stress of varying duration, rapidity of onset, and intensity, have to balance their expenditure on growth and proliferation versus stress protection. To a large degree dependent on the time scale of stress exposure, the different levels of gene expression control: transcriptional, post-transcriptional, and post-translational, will be engaged in stress responses. The post-transcriptional level is appropriate for minute-scale responses to transient stress, and for recovery upon return to normal conditions. The turnover rate, translational activity, covalent modifications, and subcellular localisation of RNA species are regulated under stress by multiple cellular pathways. The interplay between these pathways is required to achieve the appropriate signalling intensity and prevent undue triggering of stress-activated pathways at low stress levels, avoid overshoot, and down-regulate the response in a timely fashion. As much of our understanding of post-transcriptional regulation has been gained in yeast, this review is written with a yeast bias, but attempts to generalise to other eukaryotes. It summarises aspects of how post-transcriptional events in eukaryotes mitigate short-term environmental stresses, and how different pathways interact to optimise the stress response under shifting external conditions.

## Teaser

The authors review how different regulatory pathways on the RNA level interact to allow eukaryotic cells to survive and proliferate under stress conditions.

## Abbreviations

ARE: AU-rich element
CESR: Core environmental stress response
eEF: Eukaryotic translation elongation factor
eIF: Eukaryotic translation initiation factor
EJC: Exon junction complex
ER: Endoplasmic reticulum
IDR: Intrinsically disordered domain
IRES: Internal ribosomal entry site
MAPK: Mitogen-activated protein kinase
mRNP: Messenger ribonucleoprotein
mRNA: Messenger RNA
NMD: Nonsense-mediated decay
ORF: Open reading frame
PB: Processing body
PERK: Protein kinase R-like endoplasmic reticulum kinase
PTC: Premature stop codon
RBP: RNA-binding protein
RiBi: Ribosome biogenesis
RIDD: Regulated Ire1-dependent degradation
ROS: Reactive oxygen species
RP: Ribosomal protein
rRNA: Ribosomal RNA
SG: Stress granule
snRNA: Small nuclear RNA
tRNA: Transfer RNA
uORF: Upstream open reading frame
UPR: Unfolded protein response
UTR: Untranslated region

## Introduction

**Figure 1.**
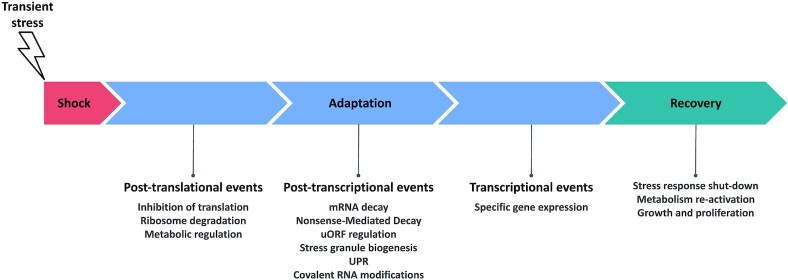
Approximate time scale and order of three layers of regulation; post-translational, post-transcriptional and transcriptional. During the eukaryotic response to transient stress, the shock, adaptation, and recovery phases are differentially composed of post-translational, post-transcriptional, and transcriptional events. During the shock phase, the cell encounters external stress and activates responses for survival. In the adaptation phase, the cell is activating stress responses and is reprogramming gene expression on different levels. The recovery phase starts when the cell is able to restart growth and proliferation, either as a result of the cessation of the external stress or because activation of the stress response has mitigated the cellular consequences of stress. The timing of the post-transcriptional level is intermediate between the fast post-translational and the slower transcriptional responses. Different types of post-transcriptional regulation are named in the figure.

Environmental stress (e.g. heat, cold, hyper- or hypo-osmosis, oxidative stress) may require immediate reactions in the cell to ensure survival (Proft and Struhl 2004). The cause of the stress has to be eliminated or reduced, and the cellular damage repaired. In a slightly longer perspective, the stress means a major drain on energy resources, forcing the cell to adapt its gene expression program (Warner 1999). Finally, in the recovery phase the cellular resources can be redirected towards growth and proliferation (Gasch *et al*. 2000, Causton *et al*. 2001, Proft and Struhl 2004). The cellular responses act on different time scales; post-translational events with pre-existing proteins occur within seconds, post-transcriptional events within a few minutes (McCarthy 1998), while transcriptional induction and repression are slower and more long-lasting (Gasch *et al*. 2000, Causton *et al*. 2001). It follows that responses on these three levels are differently suited to deal with the initial, adaptation and recovery phases of the stress response. The post-transcriptional regulation level arguably has the most fitting time scale to deal with the adaptation and recovery phases of transient stress, which typically take place in a time scale of minutes (Fig. 1).

## Core or specific response to transient stress

Cells encounter stress on different time scales and intensities. As an immediate reaction to a sudden environmental shock, cells will mount the ‘core environmental stress response’ (CESR) (Gasch *et al*. 2000, Causton *et al*. 2001), a generic transcriptional induction programme that is largely constant irrespective of the type of stress applied. As a result of the CESR, genes encoding components of the translation machinery, ribosomal proteins (RP)s and the RiBi regulon, controlling ribosome synthesis (Jorgensen *et al*. 2004), are repressed; whereas genes for carbohydrate metabolism, protein folding, and defence against oxidative stress are upregulated (Gasch *et al*. 2000, Causton *et al*. 2001). It is thought that the CESR will protect the cell against common adverse effects of stress, such as energy deprivation. Once molecular markers are in place that can inform the cell of the type of stress, the response can get more diversified and adapted to the appropriate stress type. The existence of the CESR may explain hormesis, the effect that prior exposure to low level stress may protect the cell against a second wave of stress, and also cross-resistance to other stress types (Semchyshyn 2014). It is interesting to ask whether the CESR is more extensive in immobile organisms, plants and fungi, which are unable to physically move away from sudden stress and have to rely more on intrinsic stress responses.

## Lack of correlation between transcript induction and requirement for short-term stress resistance

It has long been noted that the set of genes induced by a certain stress, often called ‘stress response genes’, is not the same as the set required for resistance to the same stress. For instance, in *S. cerevisiae* only a small fraction of the genes induced by shift to a new stressful set of conditions are required for optimal growth in those conditions (Giaever *et al*. 2002), and the genes transcriptionally induced by DNA-damaging agents have no significant overlap with the genes protecting against DNA damage (Birrell *et al*. 2002). No correlation was seen between *S. cerevisiae* genes induced by oxidative stress and those important for resistance to the same stress (Thorpe *et al*. 2004). In *Arabidopsis*, only 43 out of 16 000 genes induced by heat stress had any measurable adaptive value (Swindell *et al*. 2007). There are principally different explanations for this discrepancy. First, many of the induced genes may simply be irrelevant for survival or tolerance to stress, potentially since they are part of a stereotypic CESR that is not adapted to the specific type of stress. Second, expression of many genes together may be required for the stress protective effect, making it difficult to measure the phenotypic effect by knocking out or overexpressing one gene at a time. Third, transcriptional induction may be required for later phases of the stress responses, such as adaptation and recovery, while many gene products necessary for survival of stress need to be present already at the onset of stress or very soon thereafter, *e.g*. DNA repair proteins (Birrell *et al*. 2002). These observations indicate that we need to look beyond the transcriptional stress response to understand how cells survive the initial shock and recover after transient environmental stress.

A case in point is the *S. cerevisiae* HOG pathway, required for resistance to hyperosmotic stress. As for many other stress resistance pathways, work on this pathway has focused on regulation of transcriptional induction of target genes. However, it turns out that other mechanisms are more important for survival of the shock phase. Notably, in a clever experiment Hog1 was C-terminally tagged with a CAAX motif, anchoring it in the plasma membrane with a lipid tail (Westfall *et al*. 2008). This modified Hog1 was catalytically active but unable to enter the nucleus and induce the osmotic stress transcriptional program. Nevertheless, yeast cells carrying only the cytoplasmic Hog1 version showed equal viability after hyperosmotic shock as the wild-type, ruling out any requirement of transcription for survival of hyperosmotic shock.

Therefore, it is important to address the relevance of post-transcriptional regulation in the stress response. These mechanisms act on the adaptation and recovery phase, and in many cases are essential for stress resistance. Here, we will discuss different post-transcriptional mechanisms essential for the stress response and the interplay that can occur between them.

## Translation regulation under transient stress

**Figure 2.**
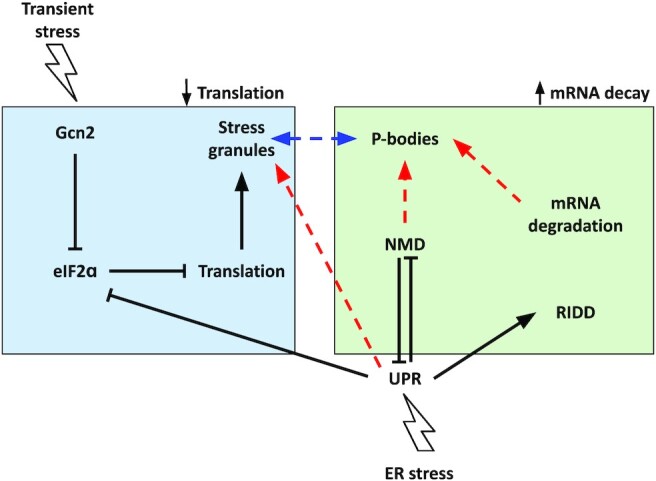
Interplay between pathways related to the eukaryotic cellular stress response. Different post-transcriptional mechanisms are activated in response to stress to regulate the energy spent in some cellular processes and in doing so focus more resources into survival and stress-resistance mechanisms. As such, the translation rate can be modulated by either inhibiting translation (blue box) or increasing degradation of mRNAs (green box). Translation initiation can be blocked by phosphorylation of the initiation factor eIF2ɑ, in response to activation of the kinase Gcn2. In such conditions, after translation is inhibited, the formation of stress-associated granules (i.e. SGs and PBs) is increased. SGs have been associated with translation silencing and PBs with mRNA degradation. They share structural components and can also exchange components in certain conditions (dashed blue arrow). On the other hand, mRNA degradation can occur through the canonical degradation machinery (i.e. exosomes or Xrn1-dependent degradation) or through targeted degradation pathways such as NMD and RIDD. Additionally, the UPR, activated in response to ER stress, can modulate post-transcriptional mechanisms involved in both mRNA degradation (RIDD activation) and translation inhibition (eIF2ɑ phosphorylation). Notably, the UPR and NMD mutually inhibit each other, allowing high and stable UPR activation under strong stress, and also providing a way to shut down the UPR once the stress has disappeared. Components of some complexes can physically interact with other signalling components (dashed red arrow), potentially helping in the regulation of the stress responses.

As a response to sudden environmental stress, the cell needs to reduce its energy spending (Warner 1999). In a rapidly growing cell, protein synthesis consumes about a third of all available energy, disregarding the energy spent on ribosome synthesis (Buttgereit and Brand 1995), making it a major target for this adaptation. Within a few minutes after onset of environmental stress, translation can be reduced to a few % of its previous value (Ashe *et al*. 2000, Melamed *et al*. 2008, Kershaw *et al*. 2015). This is achieved through events on multiple levels (Fig. 2).

Global translation is drastically reprogrammed during stress, to rapidly promote production of stress-protective proteins, halt proliferation, and diminish the energy spent on protein production (Preiss *et al*. 2003, Smirnova *et al*. 2005, Shenton *et al*. 2006, Melamed *et al*. 2008, Spriggs *et al*. 2010, Lackner *et al*. 2012). The synthesis of ribosomes is controlled by the RiBi regulon (Jorgensen *et al*. 2004), which shuts down activity very fast in response to environmental stress. Additionally, existing ribosomes will be degraded if the stress persists, mainly through ubiquitin- and proteasome-dependent pathways (Kraft *et al*. 2008). Interestingly, both ribosomal assembly and degradation may be integrated through ubiquitin, as several RPs are encoded as ubiquitin fusions (Finley *et al*. 1989).

Furthermore, under stress translation rate is reduced through inhibition of translation factors, acting both on the translation initiation and elongation steps (Spriggs *et al*. 2010, Shalgi *et al*. 2013). One well characterised pathway impinging on initiation is the evolutionarily conserved protein kinase Gcn2 phosphorylating eIF2α and thereby inhibiting the GDP/GTP exchange necessary for formation of the ternary complex including the methionine-coupled initiator tRNA (Hinnebusch 2005). This is achieved through phosphorylated eIF2α acting as an inhibitor of eIF2B, its own guanine nucleotide exchange factor (GEF), thus preventing recharging of eIF2α with GTP (Kimball *et al*. 1998, Pavitt *et al*. 1998). It is reflected by translation initiation factors involved in mRNA scanning, such as eIF4A and eIF4B, dissociating from their 5′ ends, causing cessation of translation (Bresson *et al*. 2020). A second pathway involves dephosphorylation of eIF4E-binding proteins, causing them to bind to eIF4E and global translation rate to decrease (Ayuso *et al*. 2010).

However, it is clear that stress-related inhibition of translation also involves other aspects. Oxidative stress was observed to cause repression of translation through different mechanisms in *S. cerevisiae*: inhibition of translation initiation through Gcn2-dependent phosphorylation of eIF2α, Gcn2-independent translational inhibition, and slowing of translation elongation (Shenton *et al*. 2006). The latter effect is mediated in part by phosphorylation of elongation factor eEF2 in response to oxidative stress (Sanchez *et al*. 2019). On the other hand, translation of specific mRNAs that are transcriptionally upregulated under such conditions is enhanced (‘homo-directional changes’) (Preiss *et al*. 2003).

The paradigm that the sole function of Gcn2 is through inhibition of translation initiation through phosphorylation of eIF2α, and conversely that Gcn2 is required for translational inhibition upon stress (Dever *et al*. 1992, Dever *et al*. 1993) has recently been challenged (Boye and Grallert 2020). This is on the grounds that eIF2α phosphorylation in many cases is neither necessary nor sufficient for shutdown of translation. That Gcn2 can have a wider role in cellular regulation is suggested by the observation that a *gcn2* mutation also affects aspects of the UPR such as *HAC1* splicing and expression of the ER-located disulphide isomerase Pdi1 (Gast *et al*. 2021).

Certain translation factors may have a special role under stress. The translation factor eIF5A is the only protein known to have one of its lysine residues modified to hypusine (Park 2006). Although initially named as a translation initiation factor, the major function of this protein is now believed to be in elongation (Gregio *et al*. 2009, Saini *et al*. 2009, Henderson and Hershey 2011). In eIF5A-deficient mutants, formation of both processing bodies (PBs) and stress granules (SGs) is inhibited, similar to the effects of the translation elongation inhibitor cycloheximide (Gregio *et al*. 2009, Li *et al*. 2010). One of the roles of this essential protein is to ensure that translation through proline-rich stretches proceeds without significant ribosome stalling (Gutierrez *et al*. 2013). There are indications that eIF5A can play a particular role for stress tolerance. Ectopic overexpression of yeast eIF5A yielded transgenic yeast or poplar plants with increased resistance to oxidative and hyperosmotic stress (Wang *et al*. 2012). It has also been observed that eIF5A deficiency causes increased sensitivity to acetic acid stress in yeast (Cheng *et al*. 2021). Further, in *S. cerevisiae* expression of two isoforms of eIF5A is affected by oxygen and glucose stress, and deficiency of one isoform sensitises the cells to low oxygen (Barba-Aliaga *et al*. 2020).

Recently, it has also been proposed that eIF2A can drive translation initiation under conditions where eIF2α is phosphorylated and inactivated (Komar and Merrick 2020). In yeast, deletion of the *eIF2A* gene selectively affects cap-independent translation initiation from internal ribosomal entry sites (IRES) (Komar *et al*. 2005). IRES-driven initiation of translation, originally discovered for certain viral genes is also associated with genes activated during cellular stress or apoptosis (Holcik *et al*. 2000).

## RNA decay

Degradation of mRNA can occur in conjunction with or independent of translation (Muhlrad and Parker 1994, van Hoof *et al*. 2002, Doma and Parker 2006). Decay of cytoplasmic non-translating mRNAs is often initiated by shortening of the 3′ polyA tail (Daugeron *et al*. 2001, Tucker *et al*. 2001). Protein complexes interacting with these mRNAs stimulate the removal of the 5′ cap structure (Schwartz and Parker 2000). Degradation can then proceed either from the 3′ end by the exosome complex (Anderson and Parker 1998), or from the 5′ end by Xrn1-dependent decay (Hsu and Stevens 1993, Muhlrad and Parker 1994). A link between translation and mRNA half-life is revealed by the finding that transcripts with near-optimal codon composition are more stable than those with a high proportion of rare codons. Optimal codons confer a high translation rate, while rare codons, in particular in clusters, can cause ribosome pausing and stalling, which may trigger mRNA degradation (Presnyak *et al*. 2015).

Other stress response pathways can be under mRNA stability control. In *S. cerevisiae*, the RNA helicase Dhh1 and the decapping enzyme Dcp2 bind to autophagy-related transcripts and promote their degradation under nutrient-replete conditions (Hu *et al*. 2015). Under such conditions, those mRNAs are also degraded by the mRNA exonuclease Xrn1, hence mRNA decay negatively regulates autophagy (Delorme-Axford *et al*. 2018).

## NMD

Nonsense-mediated decay (NMD) was first defined in mammalian cells, as degradation of mRNAs containing premature stop (‘nonsense’) codons; for review see (Kurosaki *et al*. 2019). These can occur through point mutations, errors in transcription, and also through splicing errors. In metazoans, splicing errors are likely the major source of premature stop codons. In line with this, the first identified and now canonical activator of NMD was the exon junction complex (EJC), which forms on an mRNA after a splicing event has been completed (Le Hir *et al*. 2000). In the first translation round of an mRNA, the translating ribosome is thought to remove these EJCs. In most yeast species with their compact genomes, introns are shorter and rarer. Consequently, splicing errors less often will trigger NMD, and the role of an EJC is unclear. Nevertheless, NMD in yeast will respond to PTCs (Losson and Lacroute 1979). Other structural features in mRNAs can trigger NMD, such as 3′-UTRs that are longer than average (Kebaara and Atkin 2009), or contain GC-rich regions (Imamachi *et al*. 2017). Overlapping reading frames also make an mRNA prone to NMD activation (Torrance and Lydall 2018) as well as upstream open reading frames (uORFs) in the 5′-UTR (Gaba *et al*. 2005, Colombo *et al*. 2017) and introns in the 3′-UTR (Colombo *et al*. 2017). It has gradually emerged that also mRNAs without any obvious aberrant structural features are subject to regulated NMD. It is estimated that 10–20% of all RNA species in eukaryotes can be regulated through NMD (Mendell *et al*. 2004, Hurt *et al*. 2013). Recently, evidence for a separate ER-localised NMD machinery in mammalian cells was presented, where a protein with a role in retrograde trafficking into the ER will also recruit NMD components to mRNAs translated in the ER (Longman *et al*. 2020).

As we have seen, the rapid downregulation of translation upon onset of stress results from changes on multiple levels. In *S. cerevisiae*, one of them is a decrease of pre-mRNAs encoding RPs. It was demonstrated that this decrease after osmotic stress occurs through NMD, as it is dependent on the NMD components *UPF1*–*3* (Garre *et al*. 2013). Indeed, NMD is upregulated during osmotic stress (Kawashima *et al*. 2014), providing a way to quickly change the translational program under stress. Depletion of Upf1 increases the levels of many mRNAs encoding stress-related proteins (Tani *et al*. 2012), and it also increases eIF2α phosphorylation, indicating that impaired NMD is stressful for the cell (Oren *et al*. 2014). Binding of the Upf1 protein to RNA is enhanced by arsenite stress (Backlund *et al*. 2020).

Paradoxically, in *Schizosaccharomyces pombe* exposed to oxidative stress, upregulation of mRNAs critical for stress survival requires Upf1. Notably, the RNA-binding protein (RBP) Csx1 is required together with Upf1 for this effect (Rodríguez-Gabriel *et al*. 2006). This observation hints that the Upf1 protein may have roles independent of NMD in the stress response.

Not only degradation but also establishment of mRNA stability is important for stress responses. Stress causes stabilisation of bulk mRNA (Hilgers *et al*. 2006). Among these, certain functional groups of transcripts required for growth and proliferation are instead destabilised, e.g. the RiBi regulon or mRNAs encoding RPs, whereas other transcripts required for stress survival and recovery are further stabilised (Rodríguez-Gabriel *et al*. 2006, Molina-Navarro *et al*. 2008, Romero-Santacreu *et al*. 2009, Miller *et al*. 2011, Garre *et al*. 2013). Of the different aspects of post-transcriptional regulation, quantitative measures for RNA stability have proven to be among the most difficult to establish. Single genes can be placed under control of a regulatable promoter, and degradation measured after transcriptional shut-off. For global studies, different methods can be used to inactivate RNA polymerase, and observe the decay rates of individual RNA species. However, these often introduce artefacts; a concern common to all these methods is the complete upheaval in the cell through the arrest of global transcription. The difficulties to measure mRNA stability with such invasive techniques have recently been reviewed (Wada and Becskei 2017). When studying stress responses, these shortcomings are particularly problematic since they mean additional stress response pathways are activated, confusing interpretation. Moreover, the time resolution of these methods is insufficient to study responses with a time scale of only a few minutes. These considerations have caused researchers to look for methods that avoid disturbing the cellular function under study. Calculating the degradation rate of an RNA species indirectly from the synthesis rate and steady-state level using radioactive *in vivo* labelling is possible (Jordán-Pla *et al*. 2019). More recently, *in vivo* metabolic RNA labelling with nucleoside analogues as a more direct approach has been used; pulse-chase labelling with an analogue followed by RNA-seq makes it possible to measure decay rates through the gradual disappearance of label from RNA species. These more recent investigations yield notably lower estimates than earlier investigations of the median mRNA half-life.

## uORFs

Some mRNAs contain short uORFs in the 5′-UTR (Hinnebusch *et al*. 2016). These can be utilised as post-transcriptional regulatory devices in different ways—to attenuate or promote translation depending on the conditions (Morris and Geballe 2000, Meijer and Thomas 2002, Ruiz-Orera and Alba 2019), or to accelerate mRNA decay as triggers for NMD (Oliveira and McCarthy 1995, Ruiz-Echevarria and Peltz 2000), as previously mentioned. Different translation patterns of uORFs in *S. cerevisiae GCN4* gene under nutrient-rich or amino acid starvation regulate translation of the main ORF into the Gcn4 transcription factor responsible for expression of genes required for amino acid synthesis (Hinnebusch 1984, Abastado *et al*. 1991), a famous example of post-transcriptional control through uORFs. Later, it was shown by ribosome profiling assays that under nutrient stress in yeast, there is a global increase in translation of small ORFs, in particular in the 5′-UTRs (Ingolia *et al*. 2009). This increase is associated with a relaxation of initiation codon recognition, such that many small ORF translation events use unconventional start codons. While the *GCN4* uORFs apparently exert their effect on translation of the main ORF without any involvement from the uORF-encoded peptides, there are other cases where peptides translated from uORFs are thought to have functional role. For the *S. cerevisiae CPA1* gene, mutational analysis of its uORF indicate that the peptide sequence of its translational product, rather than its nucleotide sequence context, are important for its effect on translation of the *CPA1* main ORF (Delbecq *et al*. 1994). An important question is what role, if any, is played by the peptides produced by the wide-spread translation under stress conditions from outside of main ORFs (Ingolia *et al*. 2009, Ingolia *et al*. 2014). Such peptides or short proteins may be important for stress responses on many levels (Schlesinger and Elsasser 2022), for instance as part of PBs (D'Lima *et al*. 2017).

## Stress-associated granules

In a eukaryotic cell, a number of nuclear or cytoplasmic membrane-less granules containing mRNA and proteins can form (Anderson and Kedersha 2006, Tian *et al*. 2020). Two of these messenger ribonucleoprotein, processing bodies (PBs) and stress granules (SGs), are particularly associated with stress (Kedersha *et al*. 1999, Buchan *et al*. 2008, Ramachandran *et al*. 2011, Guzikowski *et al*. 2019). Both are cytoplasmic, and increase in number upon a wide variety of stress conditions. While SGs are only present during relatively severe stress, PBs may exist also under unstressed conditions but increase in size and number under stress (Protter and Parker 2016, Luo *et al*. 2018). A functional relationship between PBs and SGs has long seemed plausible. It has been reported that the presence of PBs increases SG formation in *S. cerevisiae* (Buchan *et al*. 2008). The proteomes of PBs and SGs are non-identical; PBs are distinguished by containing mRNA decapping proteins, whereas SGs contain translation initiation factors and components of the 40S light ribosomal subunit, although there is significant overlap (Buchan and Parker 2009, Jain *et al*. 2016, Hubstenberger *et al*. 2017). The proteins contained in both granules are dominated by RBPs, largely factors implicated in translation initiation, mRNA decay or silencing (Hubstenberger *et al*. 2017, Markmiller *et al*. 2018, Youn *et al*. 2018). It has been speculated that SGs and PBs may dynamically exchange components. They both lack membranes, are made up by hydrophilic molecules, and have a fluid liquid droplet consistency. Moreover, they can often be microscopically observed in physical proximity (Kedersha *et al*. 2005, Kershaw *et al*. 2021).

Certain mRNAs avoid granules, e.g. those encoding heat shock proteins and stay delocalised in the cytoplasm during stress (Lavut and Raveh 2012, Zid and O'Shea 2014). Non-coding RNAs are underrepresented in PBs (Hubstenberger *et al*. 2017), however searches for sequence motifs directing RNA species to PBs or SGs have been largely unsuccessful. Special cases do exist, however; mRNAs carrying AU-rich elements (AREs), as well as longer mRNAs are targeted to SGs during ER stress (Namkoong *et al*. 2018). The traditional view of the function of PBs and SGs holds that PBs are sites for mRNA decay, while SGs serve to temporarily silence translation of growth-related mRNAs during stress by sequestering them, thus reducing energy expenditure. This view of PB and SG function has recently been challenged by the finding that only a minority (10%–20%) of all mRNA species reside in these granules even under stress conditions with a general translational shutdown (Hubstenberger *et al*. 2017, Namkoong *et al*. 2018).

There is circumstantial evidence for a role of SGs in stress survival and recovery. Overexpressing Pab1 in *S. cerevisiae* increases both the number of SGs and resistance to various stressors including acetic acid (Martani *et al*. 2015). Inhibiting SG formation by expressing a dominant-negative allele of eIF2α sensitised neuroblastoma cells to genotoxic agents (Vilas-Boas *et al*. 2016), and expression of a phosphomimetic allele of eIF4E increased both SG number and resistance to oxidative stress (Martínez *et al*. 2015). Under hypoxic conditions, SGs accumulate in human cells. The signalling scaffold RACK1 is then sequestered in SGs, leading to increased survival upon exposure to genotoxins by preventing activation of apoptosis through the MTK1 pathway (Arimoto *et al*. 2008). The underlying difficulty in interpreting these types of studies is that all these genetic alterations affect other cellular functions beside SG formation, since no gene product is uniquely involved in SGs.

PBs were initially interpreted as mRNA decay centres as they contain mRNA degradation factors, and as PBs accumulated in RNA decay mutants (Sheth and Parker 2003). The NMD pathway component Upf1 has an indirect physical interaction with the decapping protein Dcp2, a component of PBs. Upf1 also binds directly to the Dcp2 activators Edc3 and Pat1, which are also found in PBs (Swisher and Parker 2011). Such observations suggest that PBs are an essential element in RNA degradation. However, a role of PBs as centres of RNA degradation has been put in question. Contrary to expectation, an *S. cerevisiae* double mutant which lacks all of Edc4 and the C-terminal domain of Lsm4 and is defective in PB formation, shows global mRNA destabilisation (Huch *et al*. 2016). While NMD components may co-localise with PBs, the NMD pathway is unaffected in cells lacking PBs (Stalder and Mühlemann 2009). Silencing and degradation of mRNAs are required for PB formation, but blocking PB formation by depleting Lsm1 or Lsm3 does not affect mRNA silencing nor mRNA decay (Eulalio *et al*. 2007). Thus, it appears that mRNA silencing, NMD, and RNA decay are not dependent on PBs. In the light of these observations, one could speculate that PBs function to sequester RNA decay factors and prevent them from degrading RNA.

Beyond tasks for these granules in mRNA transactions, a role as intracellular signalling hubs has been proposed in particular for SGs. In *S. cerevisiae*, protein kinase A subunits are found in SGs (Tudisca *et al*. 2010), and members of the TORC1 signalling complex localises to SGs in heat stress (Takahara and Maeda 2012). In *Sz. pombe*, Pck2 (protein kinase C) is found in SGs under strong, but not moderate, heat stress (Kanda *et al*. 2021). Orthologues of the stress-activated MAP kinase Hog1 from *Sz. pombe*, *Pichia pastoris*, and *Candida boidinii* are sequestered in SGs under heat stress, but not *S. cerevisiae* Hog1 (Shiraishi *et al*. 2018). Such sequestration may serve the purpose of dampening signalling output from a pathway. *Sz. pombe* Pmk1, a MAP kinase downstream of Pck2, promotes accumulation of Pck2 in SGs under high heat stress, thus providing a negative feedback loop which prevents hyperactivation of the pathway (Kanda *et al*. 2021). Providing a possible link between SGs and NMD, the Upf1 kinase hSMG-1 aids in SG formation (Brown *et al*. 2011). It should be kept in mind, however, that silencing by sequestration is not the only possible effect on signalling proteins in SGs. The interior of granules may also provide local high concentrations of components that augment or change the specificity of a signalling pathway.

Many proteins in SGs are ubiquitinated, and the pattern of ubiquitination differs between stress types. It turns out that this ubiquitination is not required for formation of SGs, but for their subsequent disassembly. Interactions between RBPs increase under heat stress, and again ubiquitination is required to resolve such interactions (Maxwell *et al*. 2021). Failure to properly disassemble SGs, including the RBPs that are part of them, may result in persistent, dysfunctional SGs that contribute to pathogenesis.

## ER stress

In the ER lumen, proteins destined for export from the cell or insertion into cellular membranes are folded and post-translational modifications added. Proteins destined for export carry many modifications, e.g. glycosylation, disulphide bonds, lipid anchors (Braakman and Hebert 2013). Thus, their folding is slow since addition of modifications is a multistep process; further the modifications add steric constraints to the folding. For this process, the ER lumen contains large amounts of proteins required for folding events, including chaperones, co-chaperones, prolyl hydroxylases, and oxidoreductases (Braakman and Hebert 2013, Ellgaard *et al*. 2016).

The folding capacity of the ER can be exceeded under different conditions including environmental stresses such as heat, osmotic or oxidative stress; changes in physiological conditions, e.g. perturbation of Ca^2+^ homeostasis or lipid metabolism, and blockade of post-translational modifications, as well as in some pathological stresses, as in the presence of mutated proteins (Lin *et al*. 2008). In biotechnological settings, this can also happen through artificial overproduction of a secretory protein (Hussain *et al*. 2014). These conditions can cause an imbalance between the folding machinery present in the ER and the amount of substrate proteins, causing an accumulation of unfolded proteins in the ER lumen, a state known as ER stress. The Unfolded Protein Response (UPR) is a conserved signalling pathway which relieves the ER stress through increased production of proteins involved in lipid synthesis, protein folding and modification, ER biogenesis, and degradation (Walter and Ron 2011). Additionally, other responses are activated to contain with the ER stress, such as translation inhibition to decrease the ER load and degradation of misfolded proteins through the Endoplasmic Reticulum-Associated Degradation (ERAD) pathway which retro-translocates unfolded proteins to the cytoplasm where they are targeted for ubiquitin-dependent degradation (Thibault and Ng 2012).

In mammals, two of the three branches of the UPR directly involve post-transcriptional regulation. These branches are activated by ER-resident sensors that detect the presence of unfolded proteins in the lumen of the ER. First, IRE1, the most conserved UPR branch, acts through unconventional splicing of the transcription factor XBP1 governing expression of proteins required for the UPR (Yoshida *et al*. 2001). The ER stress sensor Ire1 has an endonuclease domain, which early upon activation removes a translation-blocking intron from the *XBP1* mRNA, thereby allowing efficient translation of Xbp1 (Yoshida *et al*. 2001, Calfon *et al*. 2002, Lee *et al*. 2002). Later in the stress response, Ire1 will also cleave mRNAs to decrease the overall translation load in the ER (Hollien and Weissman 2006, Han *et al*. 2009, Hollien *et al*. 2009). Second, through the ER membrane-bound protein kinase PERK phosphorylating eIF2α, leading to global downregulation of translation (Harding *et al*. 1999). In *S. cerevisiae*, the Ire1-mediated unconventional splicing of an mRNA encoding a transcription factor as part of the UPR is conserved. There, Hac1 has this role, and Ire1 removes an intron from the *HAC1* mRNA, promoting efficient translation of Hac1 protein (Sidrauski and Walter 1997). In mammals, an additional mechanism in the Ire1 branch is active. The regulated Ire1-dependent decay (RIDD) pathway will promote degradation of certain mRNAs localised in the ER (Hollien and Weissman 2006), whereas other mRNAs are instead stabilised. Interestingly, this branch is present in *Sz. pombe* (Kimmig *et al*. 2012), but absent in budding yeast.

It is presently unclear if and how cytoplasmic SGs and PBs communicate with UPR and the ER lumen. Recent observations hint at physical connections. Tubular extensions of the ER are in close proximity with PBs and may represent sites of PB and SG fission, and potentially contacts between these two granules. Inhibition of translation led to increased ER-PB contacts, while ER stress had the opposite effect (Lee *et al*. 2020). Additionally, recruitment of selected ER-targeted mRNAs into SGs in response to UPR was recently reported, suggesting a mechanism where SGs can act in the stress response as storage for newly synthesised transcripts (Child *et al*. 2021).

## The epitranscriptome under stress

Only recently, the study has begun of how RNA modifications may affect the stress response, and as yet we do not have a coherent picture. Covalent modifications of RNA molecules do occur under stress. In fission yeast, translation of stress-related genes during oxidative stress is facilitated by a covalent modification (mcm^5^U_34_) of tRNAs recognising codons that are over-represented in such genes (Fernández-Vázquez *et al*. 2013). Across several budding yeast species, the levels of the s^2^U_34_ modification increase in tRNAs during heat stress (Alings *et al*. 2015). Also in other RNA classes, stress-related modifications occur. The profile of pseudouridine modifications in mRNA and rRNA interestingly changes upon starvation (Carlile *et al*. 2014). In diverse eukaryotic organisms, N^6^-methyladenosine (m^6^A) is an abundant modification. It has been associated with alterations in transcript stability, nuclear export, translation, and splicing in different studies (He and He 2021). Under heat stress in mammalian cells, a subset of mRNAs carry increased levels of m^6^A in their 5′-UTRs, which allows them to be translated by cap-independent mechanisms and so escaping the global suppression of cap-dependent translation (Zhou *et al*. 2015). Notably, proteins recognizing m^6^A are enriched in SGs, and have also been found in PBs (Guzikowski *et al*. 2019). In *S. cerevisiae*, m^6^A is found in meiotic, but not in mitotic cells except under rapamycin-induced stress (Bodi *et al*. 2015). Interestingly, the only detectable m^6^A modification in *Sz. pombe* RNA is one position of U6 snRNA, which is fully modified. A mutant lacking m^6^A is sensitive to DNA-damaging agents and salt stress (Ishigami *et al*. 2021). This mutant also exhibits broad changes in mRNA splicing patterns, and the levels of several transcripts implicated in stress resistance are depressed.

Despite the circumstantial evidence that post-transcriptional RNA modifications change during stress, the connections to established stress-related pathways remain unexplored. Given the importance of RNA modifications for the innate immune response (Li and Rana 2022), it is conceivable that they could also function as markers for cellular stress.

## Importance of moderating the stress response

The cell must be able to modulate the stress response and shut it down after the stress agent is gone. This means striking a fine balance between growth and protection against stress. Our view of the stress response is biased by the artificial laboratory settings, where typically stress is applied either with a sharp increase up to a defined threshold (‘transient stress’), or at a constant level for long periods of time (‘chronic stress’). Life outside of the laboratory offers more complex challenges, however. An organism may be exposed to fluctuations of stressors of different amplitude and frequency. To remain competitive in the population, it needs to respond adequately in order to maintain maximal viability and reproductive fitness. For this, the time of onset, as well as rate of increase and decrease of the stress response has to be tightly controlled. It is also vital not to initiate a stress response if the stress level is too low, which can be ensured by a threshold mechanism. Finally, there has to be a way to recover after stress, and resume growth with as little delay as possible.

An interesting concept in signalling dynamics was put forward in studies of the trade-offs of signalling accuracy vs. response time of the *S. cerevisiae* HOG pathway in its response to hyperosmotic stress (Granados *et al*. 2017). In this pathway, two upstream branches feed signals to the MAPK cascade. The authors show that the Sln1 phosphorelay branch is fast to activate, whereas the response from the slower Ste11 branch provides a more accurate signalling level, leading to restoration of cell volume. Using ramped levels of environmental stress factors, the authors conclude that the fast Sln1 branch responds to the time derivative (slope) of the stress level. In a mutant with only the Sln1 branch active, and lacking the slow Ste11 branch, Hog1 signalling overshoots. This indicates that the slow branch is essential for tuning the response level. The separation between sensing an absolute stress level and a rate was corroborated in a later study, where it was found that there is a lower rate threshold under which the pathway does not activate, and that this is dependent on the Hog1 phosphatase Ptp2 (Johnson *et al*. 2021). The establishment of a lower stress threshold is important to keep noise from prematurely activating stress responses. In *S. cerevisiae* mutants lacking components of the mRNA-binding Lsm1–7/Pat1 complex, the activation threshold for hyperosmotic stress for induction of stress-activated proteins is lowered. This indicates that this complex may serve to dampen the translational response to low levels of osmotically active molecules (Garre *et al*. 2018).

The NMD and UPR pathways are mutual antagonists on the post-transcriptional level (Goetz and Wilkinson 2017). A model has been proposed where at low ER stress levels, NMD degrades mRNAs critical for the UPR pathway (Karam *et al*. 2015), thus providing an activation threshold and preventing inappropriate triggering of the UPR. Likewise, at the end of the stress response, where the UPR has eliminated most of the causes of ER stress, NMD would serve to close down remaining UPR activity. On the other hand, when the UPR is activated at high levels, it will down-regulate NMD (Karam *et al*. 2015). This dynamic ensures a robust UPR under strong ER stress, and reduces the noise level of the UPR, as well as preventing long-lasting UPR activity. A plausible scenario is that the UPR-mediated phosphorylation of eIF2α leads to NMD inhibition: many conditions that confer eIF2α phosphorylation, such as ROS, hypoxia, and amino acid starvation, also suppress NMD (Goetz and Wilkinson 2017). The mechanisms are only partially clear, but in mammalian cells they may involve Atf4. The mRNA encoding this transcription factor and UPR effector, has two uORFs, and is an NMD target. Reduced translation of the uORFs increases translation into Atf4 protein and also reduces NMD-mediated degradation of the *ATF4* mRNA, allowing escape from NMD repression of UPR (Goetz and Wilkinson 2017).

## Conclusion

The control mechanisms for stress responses have to be sophisticated to avoid detrimental effects on the cell. They should not be triggered too easily, in order not to frequently arrest cell growth and proliferation. At the same time, they have to be activated with sufficient speed and amplitude, in order to prevent cell damage, and subsequently be tuned down for the recovery phase. For some of these controls, the post-transcriptional level has the most appropriate time scale. RBPs play a major signal transducing role in stress-dependent post-transcriptional control. The importance of covalent RNA modifications in the post-transcriptional stress response, and their relation to RBPs, is only beginning to unravel.

To achieve these goals, different stress response pathways acting on translation and RNA decay have to be coordinated. NMD is now realised to have a much wider range of targets than originally thought. It can act as a moderator of other stress-related pathways, as exemplified here for the UPR. Stress-associated RNA granules have long been seen as repositories for silencing or degrading RNAs that are not needed during stress. With the knowledge that only a minority of each RNA species is contained in granules even under stress, we have to modify our thinking of how these granules affect the cell, from depleting RNAs from the free cytoplasm, to include the possibility that they actively affect their surroundings. Local high concentrations in granules of *e.g*. signalling or RNA-modifying proteins, or distinct physicochemical environments, may favour other reactions than the free cytoplasm. For instance, recent microscopic findings indicate possible connections between cytoplasmic SGs and UPR signalling originating from the ER, an example of the association between compartments and signalling pathways in the stress response.
